# Automatic detection of ictal activity in EEG using synchronization and chaos-based attributes

**DOI:** 10.1007/s11517-023-02916-w

**Published:** 2023-09-07

**Authors:** Asma Mahgoub, Marwa Qaraqe

**Affiliations:** 1 Hamad Bin Khalifa University, Doha, Qatar; 2 Present Address: Qatar University, Doha, Qatar

**Keywords:** Electroencephalography, Entropy, Neuronal synchrony, Seizures, Support vector machine

## Abstract

Automatic seizure onset detectors (SODs) have been proposed to alert epileptic patients when a seizure is about to happen and in turn improve their quality of life. Yet, the detectors proposed in literature are complex and difficult to implement in real-time as they utilize large feature sets with redundant and irrelevant features. Hence, the aim of this work is to propose a simple and lightweight SOD that exploits two characteristics that reflect the neuronal behavior during a seizure. Namely, the synchronization between EEG channels and the chaoticity of the EEG; synchronization was measured by the condition number while the recurrence period density entropy estimated the chaoticity of an EEG signal. A support vector machine was trained and tested on 10 patients from a scalp EEG dataset and was able to detect the considered seizures with a sensitivity of 100% and a false positives rate of 0.5 per hour. The results indicate that synchronization and chaos attributes can reflect the manifestation of seizures in EEG data and can be used to develop SODs. This work emphasizes that even a single relevant feature can produce an SOD with comparable performance to SODs that use many features.

**Graphical Abstract:**

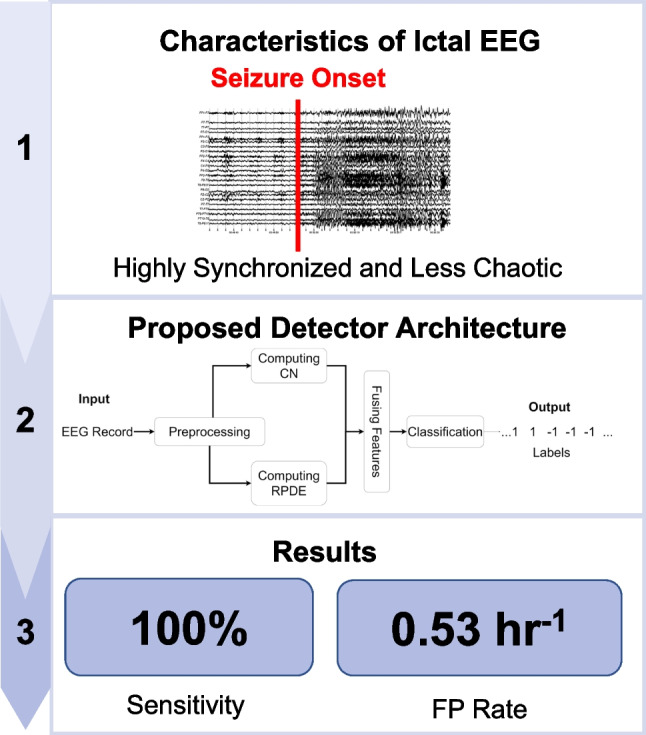

## Introduction

Epilepsy is a chronic neurological disorder that affects more than 50 million people worldwide [1]. Epileptic patients are at risk of experiencing recurrent seizures, where a seizure is a transient occurrence of symptoms that may include convulsive activity or loss of consciousness [2]. Unfortunately, till date, epilepsy cannot be treated completely, but it can be managed by anti-epileptic drugs or brain surgery. Doctors can detect seizures, diagnose epilepsy, and determine its type by using electroencephalography—a technique that uses electrodes situated on the scalp or brain cortex to record the underlying electrical activity of the brain in an electroencephalogram (EEG) record [3]. The process of scanning long-term EEG records is tiring and can be prone to human-based errors. Automatic seizure onset detectors (SODs) have been proposed to reduce the burden on doctors and to improve the quality of life of epileptic patients by alerting them when a seizure is about to happen.

There has been significant effort in the literature dedicated to the development of SODs. Many researchers consider the usage of EEG to develop SODs because of the robustness of the EEG signal in carrying ictal activity (activity belonging to a seizure). The development of an EEG-based SOD is composed of two main stages: feature extraction and classification. Different types of features can be extracted from the EEG signal for seizure detection.

Time domain features are extracted directly from the EEG signal. Many of these features were used for seizure detection. These include signal’s amplitude [4, 5], statistical features [6], and nonlinear features such as line length [7], Lyapunov exponents [8, 9], entropy [10], and synchronization [11, 12]. Time-domain features are generally simple to extract, but they are influenced heavily by the presence of artifacts in the EEG signal.

Other SODs utilized frequency-based features; these features are obtained after filtering the signal or transforming it to its frequency spectrum by using Fourier transform or any of its variants. As different seizure types are associated with different frequency bands, the usage of frequency-based features was popular in many of the previous works. Spectral features were used either by themselves [13, 14] or combined with other features [15, 16].

Wavelet-based features can capture both temporal and spectral information of a signal, and they are suitable for nonstationary signals. A signal is converted to a set of coefficients using wavelet transform, and these coefficients can be used as features by summarizing them in the form of statistical measures [17] or estimating their energy [18, 19], entropy [20–22], or even using them to quantify nonlinear measures after enhancing them by common spatial pattern (CSP) and principal component analysis (PCA) [23]. In summary, wavelet-based features are powerful and attractive, but using them usually leads to a high number of features, and this may eventually lead to overfitting.

Besides these features, some of the recent works used the raw EEG signals directly. Techniques like convolutional neural networks (CNNs) enabled the processing, feature extraction, and classification of EEG data. A channel-restricted CNN was used in [24] to detect seizures; the convolutions were adjusted to only occur between samples belonging to the same channel. Other works like [25] utilized some enhancement techniques on the signals before feeding them to the network. However, CNNs have multiple trainable parameters, and their models are hard to interpret.

After reviewing the literature, it was noted that most papers focused on optimizing the classifier’s performance rather than selecting a feature that can identify an ictal stage. In fact, the most crucial part of developing an automatic SOD is the selection of a suitable feature set [26]. Most of the discussed papers presented a set of features with a little focus on explaining how each individual feature is useful in detecting seizures. Focusing on identifying the relevant features is useful in developing an explainable machine learning model, which is crucial in medical applications. Most of the investigated work uses transformations and/or linear features. Mathematical transformations are associated with a higher computational load, and the use of linear features does not capture the dynamics of the complex activity of the brain adequately. Additionally, many of the works [13–16, 19–25] utilized a large feature set for training and detection, which is not efficient. Hence, in this work, the focus is on investigating time-domain, nonlinear features in developing an SOD with low computational complexity.

The emergence of less chaotic and synchronized activity among the neurons indicates irregular brain activity [3]. Hence, this work proposes the use of synchronization and chaos features to detect a seizure in EEG. The condition number (CN) is used for quantifying neuronal synchrony between EEG channels, and recurrence period density entropy (RPDE) is used to quantify chaos within a channel. Both measures have potential for detecting seizures in EEG, yet they are not thoroughly investigated in the field of seizure onset detection. Our previous work in [27] demonstrated the effectiveness of CN in building a synchrony-based SOD. In this work, both the CN and RPDE are selected to develop a hybrid synchrony and chaos-based SOD. This work is part of the thesis published in [28].

The main contributions of this paper are the following:

1. Utilizing features that reflect the neuronal behavior during a seizure, namely synchronization between EEG channels and chaoticity of an EEG signal to detect seizures.
2. Demonstrating that the synchronization (quantified by CN) and chaos (quantified by RPDE) can effectively differentiate between seizure and non-seizure EEG.
3. Demonstrating that the utilized features can detect seizures individually and when fused in a classification algorithm.

## Methodology

**Fig. 1:**
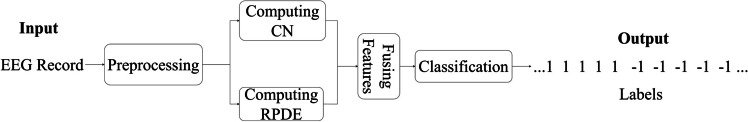
Proposed SOD architecture

The proposed SOD has the architecture shown in Fig. 1. The first component is the preprocessing stage; then, the input is segmented into epochs using a 20-s overlapping window that shifts by 1 s. For each epoch, the CN and RPDE are computed and concatenated in one feature vector. After that, a support vector machine (SVM) classifier is used to classify ictal and non-ictal EEG epochs. The following sections provide detailed description of the SOD’s stages.

### Dataset

The developed SOD is built using the CHB-MIT scalp EEG dataset [29, 30]. The dataset was produced at the Children’s Hospital Boston, and it consists of scalp EEG records of 24 pediatric patients with drug-resistant seizures. The EEG records were recorded by placing electrodes on the scalp following the international 10–20 system of electrode positioning. A bipolar montage was used to produce a multi-channel EEG signal consisting of 23 channels per record. Each channel reflects the voltage difference between two adjacent electrodes. In this study, the EEG records of 10 patients are used; most of the patients are females, and their ages are in the range of 3 to 19 years old; each patient experienced at least 3 seizures. The other patients were excluded either due to being less than 3 years old, using a different montage, having less than 3 seizure-containing records or having highly noisy EEG records. The signals had a sampling rate of 256 samples per second with a resolution of 16 bits. In this study, we use about 100 h of EEG with 60 seizures.

### Preprocessing

The first stage of the proposed SOD is preprocessing to rid the data of noise and redundancy. The identical or highly correlated channels are removed from the EEG records; at the end, 19 channels out of 23 are used in this study. Low pass filtering is implemented to remove high-frequency artifacts. A cut-off frequency of 40 Hz is chosen as most of the ictal activity occurs at frequencies below 40 Hz [31]. Additionally, the mean of each EEG record is subtracted to remove any DC offset. Finally, to produce better classification results, each signal of an EEG record is normalized to scale the signal’s range to be within [-1,1].

### Feature extraction

Determining the characteristics of ictal EEG is crucial before building an automatic SOD. In the proposed SOD, synchrony and chaos features are used for seizure detection. In this section, the process of calculating the CN and RPDE and the construction of the feature vector is explained.

#### Synchrony

The first extracted feature is the synchronization between EEG channels. Synchronization is an appearance of some relations between functionals of two processes due to interaction [32]. Synchronization can be quantified by the CN, a measure of the ill-conditioning of a system that can also reflect the synchrony between signals. In this work, the CN is chosen because it is a simple measure that can directly estimate the synchronization between multiple signals. A high CN value indicates that the signals are highly synchronized with each other. CN for the current EEG epoch (denoted by matrix $E$) is obtained using

1 $$\mathrm{CN}=\frac{{\sigma }_{\mathrm{max}}}{{\sigma }_{\mathrm{min}}}$$

where ${\sigma }_{\mathrm{max}}$ and ${\sigma }_{\mathrm{min}}$ are the maximum and minimum singular values obtained from the singular value decomposition of the current epoch $E$.

Singular value decomposition decomposes a matrix intro three matrices, such as

2 $$E=U\Sigma {V}^{\mathrm{T}}$$

where $U$ is an $m \times m$ orthonormal matrix, $V$ is an $n \times n$ orthonormal matrix, and $\Sigma$ is an $m \times n$ rectangular diagonal matrix of singular values.

#### Chaos

The second extracted feature is the chaoticity of the signal. Chaos refers to a state of unpredictability in the behavior of a system. In this work, chaos is quantified by RPDE which has a value between 0 and 1, where 0 indicates a periodic signal, and 1 indicates a random signal [33]. During a seizure, the RPDE value approaches 0 as the EEG channel becomes more organized and less chaotic. In this implementation, only the first second of the extracted epoch $E$ is used for calculating the RPDE. RPDE is measured per channel in two main steps: converting the signal of an EEG channel to a series of phase space vectors and quantifying the recurrence periods within the constructed phase space.

Firstly, a single EEG channel signal $s$ (a column vector) is converted to its phase space representation $S$ by using time-delay embedding approach and it results in the following matrix:

3 $$S=\left[\begin{array}{cc}\begin{array}{cc}s& {s}_{\tau }\end{array}& \begin{array}{cc}\cdots & {s}_{\left(d-1\right)\tau }\end{array}\end{array}\right]$$

where $s$ is the original signal and it starts at $t=0$, signal ${s}_{\tau }$ starts at $t=\tau$, and so on. The parameter $\tau$ represents the time delay, in seconds, between the elements within the sequence, and $d$ is the embedding dimension and represents the dimension of the phase space. In this work, $\tau =0.02 s$ and $d=3$, and these values were determined experimentally.

Then, around each point ${S}_{i}$ (a row in $S$), a ball with radius $\varepsilon$ is formed, in this implementation $\varepsilon =0.01$. A trajectory is followed forward in time by visiting the subsequent points, and every time the time series returns to this ball after leaving, it is referred to as the recurrence time *T*. This time is recorded in a histogram $R(T)$. The histogram is normalized to obtain the recurrence time probability density $P(T)$:

4 $$P\left(T\right)=\frac{R(T)}{\sum_{i=1}^{{T}_{\mathrm{max}}}R(i)}$$

where ${T}_{\mathrm{max}}$ is the maximum recorded recurrence time.

The recurrence time probability density, $P(T)$, is used to obtain the RPDE by the following equation:

5 $$\mathrm{RPDE}=\frac{-\sum_{i=1}^{{T}_{\mathrm{max}}}P\left(i\right)\mathrm\,{ln}\,P(i)}{\mathrm{ln}\,{T}_{\mathrm{max}}}$$

The above steps are used to estimate the RPDE value for a single channel in an EEG epoch. When the histogram $R(T)$ is empty, it is assumed that the $\mathrm{RPDE}=0$, and the periodicity was not captured by the current value of $\varepsilon$.The detailed steps for calculating the RPDE are given in the appendix.

#### Feature vector

**Fig. 2:**
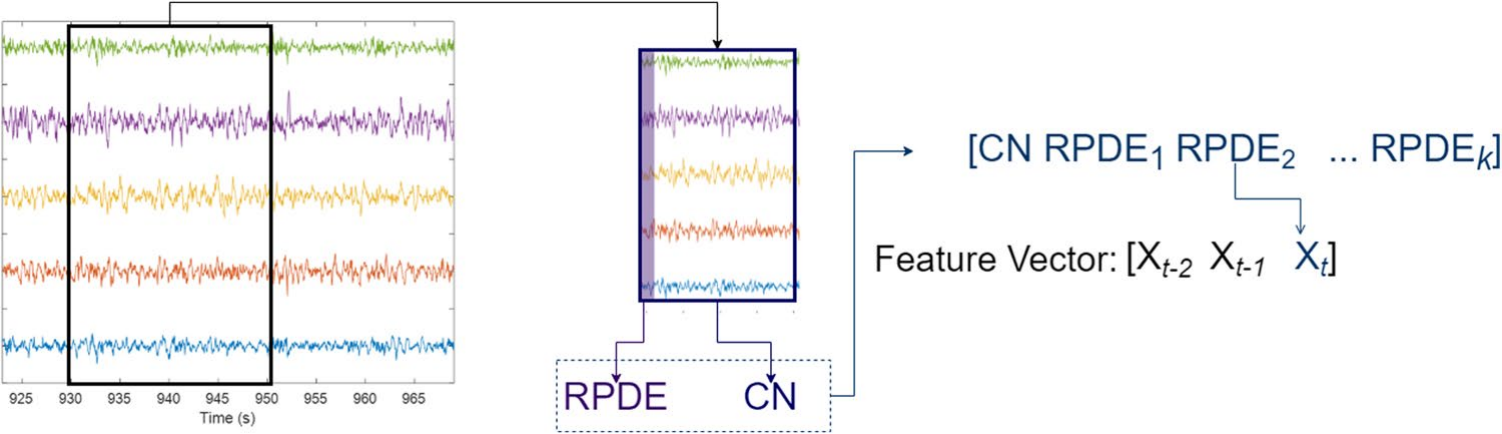
Feature extraction process overview. An EEG epoch is used to quantify the CN and RPDE. For each epoch, there is one CN value and $k$ RPDE values

The feature extraction process is illustrated in Fig. 2. Each EEG epoch is characterized by CN and RPDE. An epoch, $E$, is an $m\times n$ matrix where $m$ denotes the number of EEG channels, and $n$ is the number of time samples within the epoch. For each epoch, the CN and RPDE are computed; the features extracted from each record are normalized using *z*-score normalization. The next stage is the feature fusion stage where the extracted features are fused together in one feature vector. The feature vector captures the synchronization and chaos feature within each epoch, but it is missing any temporal information. Therefore, following the same approach as in [13], temporal features are added by concatenating the feature vectors from the previous two epochs along with the current epoch such as:

6 $$\chi = \left[\begin{array}{ccc}{X}_{t-2}& {X}_{t-1}& {X}_{t}\end{array}\right]$$

where $\chi$ is the feature vector representing an EEG epoch at time $t$ (${E}_{t}$) and it consists of vectors${X}_{t-2}, {X}_{t-1 },\mathrm{ and }{X}_{t}$. These vectors hold the CN and RPDE values of the current epoch $t$ and the previous two epochs $t-1$ and$t-2$. The vector ${X}_{t}$ within the feature vector contains the fused CN and RPDE values for an epoch ${E}_{t}$, such as $${X}_{t}=\left[\begin{array}{cc}\begin{array}{cc}\mathrm{CN}& {\mathrm{RPDE}}_{1}\end{array}& \begin{array}{cc}\dots & {\mathrm{RPDE}}_{\mathrm{k}}\end{array}\end{array}\right].$$ For each epoch, a single CN value and $k$ RPDE values are computed. In total, the feature vector $\chi$ of an EEG epoch consists of $3(k+1)$ features. The RPDE values for $k$ channels are used in the feature vector. For each patient, a record is used to observe the behavior of RPDE during a seizure in all channels; only the $k$ channels with the expected behavior are considered in the feature vector and are used to compute the RPDE values. Originally, RPDE should be computed for all 19 channels, but not all the EEG channels showed the expected RPDE behavior during a seizure; this might be due to the irrelevance of some channels to a seizure (as in focal epilepsy). The value of $k$ and the list of channels for each patient are shown in Table 1.

**Table 1:** The value of $k$ and list of channels used in RPDE calculation

Patient #	$k$	List of channels
1	6	F7T7, C3P3, P8O2, T7FT9, FT9FT10, FT10T8
3	6	F7T7, T7P7, P7O1, FP1F3, C4P4, P8O2
5	6	T7P7, P3O1, C4P4, P8O2, FZCZ, CZPZ
7	9	F7T7, P7O1, F3C3, C3P3, F4C4, C4P4, T8P8, P8O2, CZPZ
8	2	C3P3, CZPZ
9	4	C4P4, F8T8, T8P8, P8O2
10	3	T7P7, P7O1, T7FT9
19	14	F7T7, T7P7, P7O1, F3C3, C3P3, P3O1, CZPZ, F4C4, C4P4, T8P8, P8O2, T7FT9, FT9FT10, FT10T8
20	3	P3O1, T7FT9, FT10T8
24	5	F3C3, F4C4, FZCZ, T7FT9, FT10T8

### Classification

In the classification stage, the feature vector is fed into a binary SVM classifier to predict whether the current epoch is non-ictal or ictal. Binary SVM separates a set of points that belong to two classes by creating a wide-margin hyperplane between them. For linearly inseparable data, it finds the optimal soft-margin hyperplane by allowing some misclassifications to happen. The SVM hyperparameters include the value of $C$ (a regularization parameter that controls the penalty on misclassifications.), kernel’s type, and its parameters. In this work, the hyperparameters are tuned using MATLAB’s built-in “*OptimizeHyperParameters*” option. Besides the proposed synchrony and chaos SOD, two more SVM classifiers were trained and validated. In total, there were three different SODs: synchrony-based SOD, chaos-based SOD, and a hybrid synchrony and chaos-based SOD. All the used classifiers utilized a radial basis function (RBF) kernel.

The values of $C$ and $\gamma$ for each detector were chosen as follows:

1. Synchrony-based SOD: $C=0.3, \gamma =11.1$
2. Chaos-based SOD: $C=5, \gamma =0.0278$.
3. Synchrony and Chaos SOD:$C=9 ,\gamma =0.00444$.

In this implementation, a seizure is declared when at least 3 consecutive ictal labels are observed in the predicted labels. This time constraint is enforced to reduce the falsely labeled epochs. The time constraint was enforced in the synchrony and the hybrid SOD while the chaos-based SOD achieved the optimal performance when no time constraint was enforced. If a seizure is declared within the duration of an actual seizure, the alarm is regarded as a true alarm and a seizure is considered to be detected.

The proposed SOD is patient-specific; meaning that the detector is trained and evaluated on one patient at a time. For each patient, three or more seizure-containing EEG records and two normal records are used. In the training stage, all but one of the records are used for training the model; the remaining record is used for validation. This is referred to as a leave-one-record-out cross validation approach [13]. This is repeated until all seizure-containing records are tested.

## Results

**Fig. 3:**
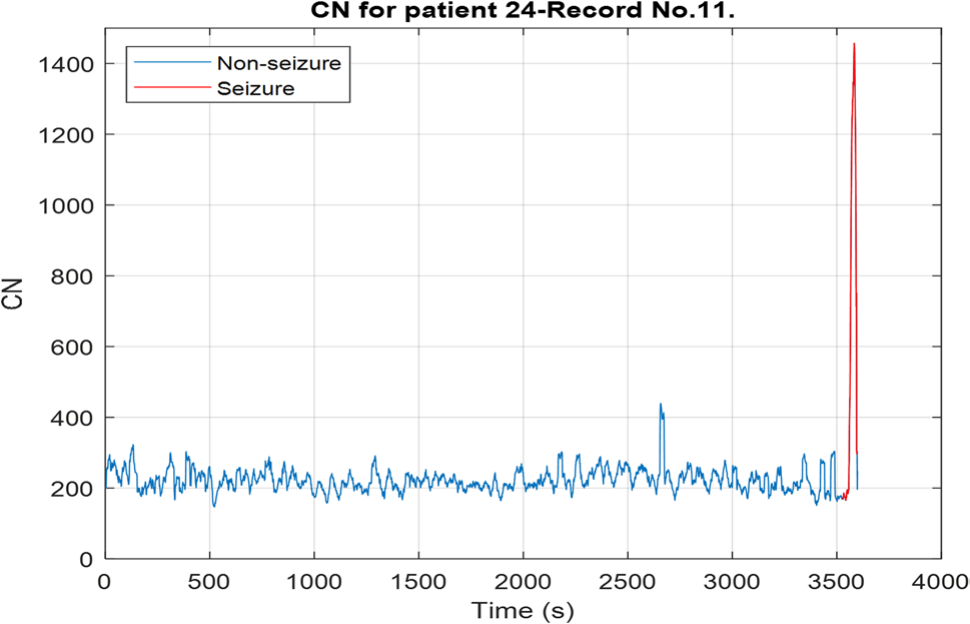
CN for the frames of an EEG record of an epileptic patient. It is evident that the CN has a significantly high value during a seizure

**Fig. 4:**
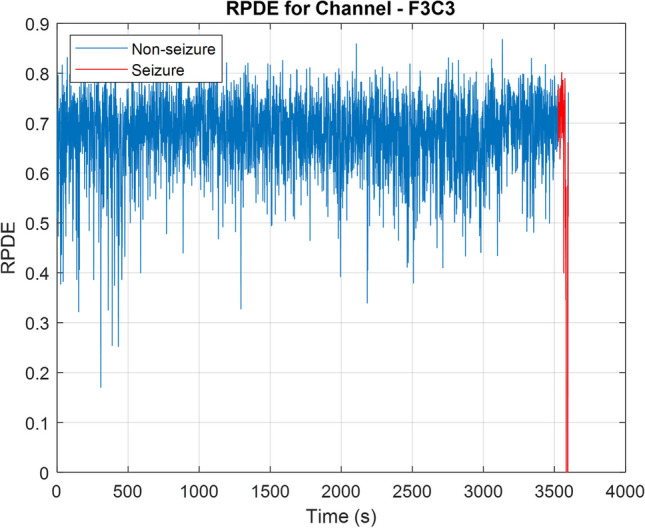
RPDE of each EEG epoch in one channel (F3C3) of a seizure-containing EEG record. The plot indicates that the values during a seizure are approaching zero

To assess the ability of CN and RPDE to distinguish between normal and ictal EEG, the CN and RPDE values of each epoch in one record are plotted. The obtained plots are shown in Fig. 3 and Fig. 4. From both figures, the great difference between seizure and non-seizure CN and RPDE values indicates that they can be used in seizure detection.

The performance of the proposed SOD is evaluated using the following metrics: sensitivity, false positive (FP) rate, and latency. Sensitivity is estimated as an event-based measure which represents the percentage of seizures that are correctly detected by the SOD:

7 $$\mathrm{Sensitivity}=\frac{\mathrm{No}.\mathrm{\;of\;detected\;seizures\;}}{\mathrm{Total\;No}.\mathrm{\;of\;seizures}}\times 100\%$$

FP rate is the number of falsely labeled epochs per 1 h, it is calculated using the below equation.

8 $$\mathrm{FP\;rate}=\frac{\mathrm{no}.\mathrm{\;of\;mislabeled\;normal\;epochs}}{\mathrm{duration\;of\;record\;}(\mathrm{hr})}$$

Latency is the duration (in seconds) between the actual onset time and the onset time declared by the SOD.

As mentioned in the previous section, 3 SODs were developed, and their performance is presented in this section. The first SOD utilizes the synchrony features; this detector uses a feature vector that consists of one feature only which is the CN. Table 2 summarizes the performance of this simple detector. All the seizures experienced by 8 out of 10 patients are successfully detected. The other seizures may have been missed due to having lower CN values compared to other seizures. Yet, for all patients, the FP rate and latency are high.

**Table 2:** Average performance of the synchrony-based and chaos-based SOD

Used feature	Sensitivity	FP rate (hr^−1^)	Latency (sec)
Synchrony (CN)	97.08%	4.70	16.67
Chaos (RPDE)	98.67%	4.00	10.53

The chaoticity of neuron activity is used by the second SOD. This chaos-based detector utilizes a feature vector consisting of $k$ RPDE values; one for each channel as per the channels presented in Table 1. Table 2 shows a summary of the performance of the chaos-based SOD. In terms of the three performance metrics, the performance of this SOD is better when compared to the synchrony-based SOD, but it is more complex as it uses more features.

**Fig. 5:**
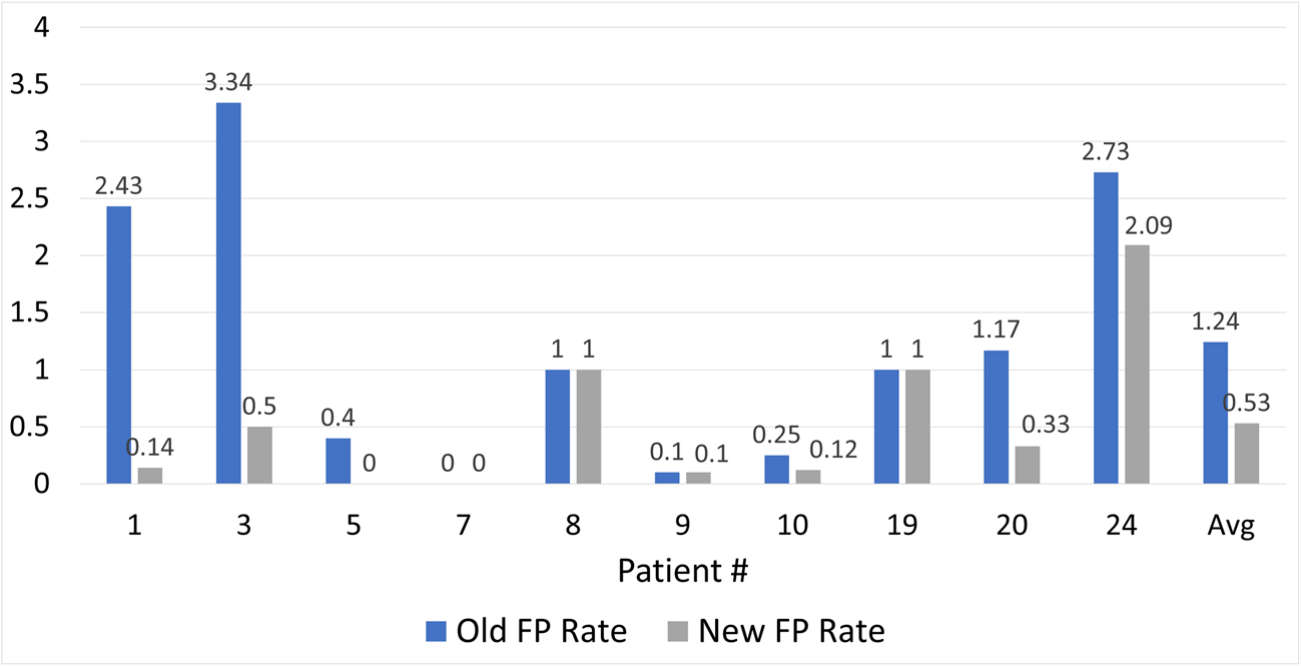
Effect of extending the duration of seizure on the FP rate. The FP rate reduced in almost all patients

In Table 3, the performance of the proposed hybrid synchrony and chaos SOD is shown. This detector uses the feature vector in (6). The results indicate that the seizures experienced by all patients were detected with 100% sensitivity while having an average FP rate of 1.24 false alarm per hour and an average onset detection latency of around 9.7 s. The average FP rate and latency are relatively high. However, many false alarms were within 30 s before or after a seizure. As this duration is very close to the seizure, these false alarms can be considered a part of the seizure. Hence, the performance evaluation algorithm was adjusted to ignore any false alarm that is declared within this time. This modification reduced the reported FP rate in 6 out of 10 patients as indicated in Fig. 5, and the average FP rate was reduced to 0.53 per hour.

**Table 3:** Performance of the synchrony and chaos SOD

Patient #	Sensitivity	FP rate (hr^−1^)	Latency (sec)
1	100%	2.43	6.00
3	100%	3.34	11.83
5	100%	0.40	9.20
7	100%	0	15.67
8	100%	1.00	6.40
9	100%	0.1	4.50
10	100%	0.25	6.25
19	100%	1.00	17.33
20	100%	1.17	11.38
24	100%	2.73	8.73
Average	100%	1.24	9.73

## Discussion

This work aims to use a limited number of features to build an SOD that detects seizures with high sensitivity and low latency; the focus is on using features that reflect the neuronal behavior during a seizure. Although it is challenging to develop an SOD that uses one feature, the results in Table 2 indicated that a single feature (synchrony or chaos) can be used to detect seizures successfully. Yet, there is a need to further improve the performance in terms of FP rate and latency.

Both synchrony and chaos have their own advantages. CN is a single value that can be used to quantify synchronization between all channels within an EEG epoch. On the other hand, RPDE measures chaoticity of a single channel. As adding more features to a machine learning model would usually improve the performance, it is expected that RPDE will have a better seizure detection performance compared to the CN. Also, RPDE only considers the EEG channels that are relevant to a seizure; this restriction improves the performance further, especially for focal seizures. Yet, calculating RPDE is complex, slow, and requires setting multiple parameters such as $\tau$,$d$, and $\epsilon$.

To further improve the performance of the SODs presented in Table 2, a hybrid synchrony and chaos SOD is proposed. Fusing synchrony and chaos features have improved the performance as indicated in Table 3. Sensitivity increased to 100%, and the FP rate is reduced by almost 90%. When both features are used, the EEG epochs that are labeled as “ictal” have both high synchronization and low chaoticity; this means that they are more likely to be actual “ictal” segments.

**Fig. 6:**
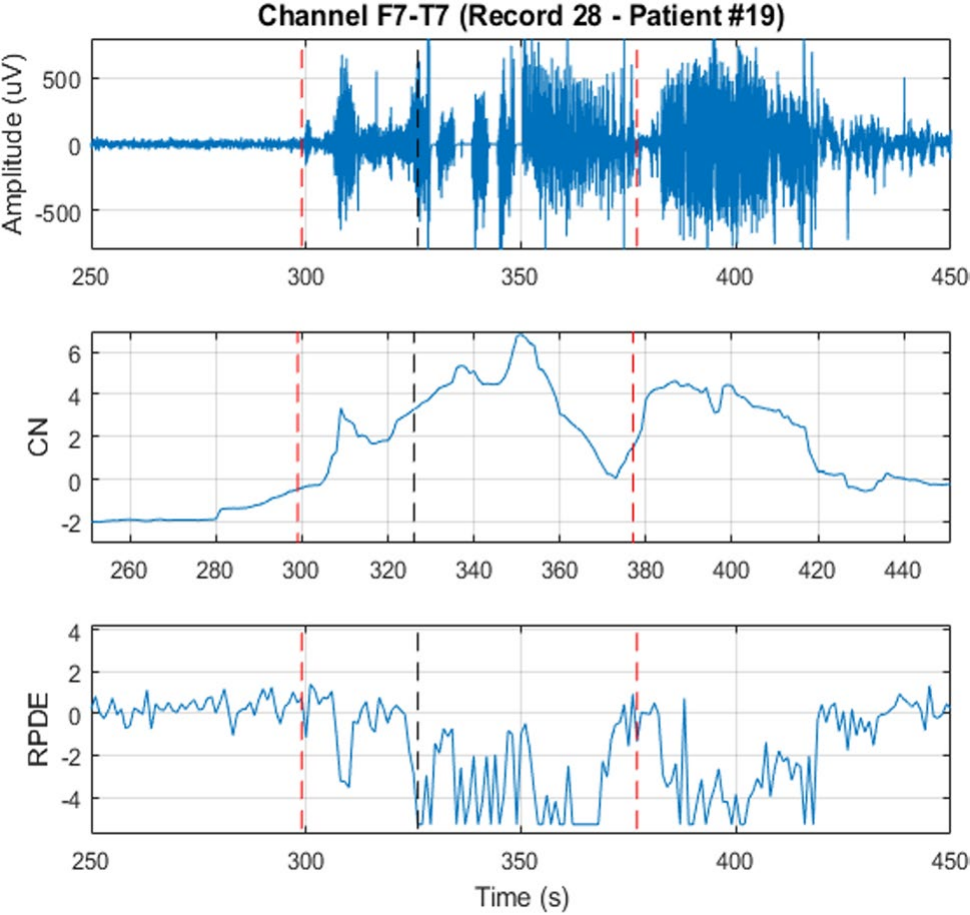
The electrical activity, CN, and RPDE of an EEG channel during a seizure. The dashed red lines indicate the seizure’s onset and offset. The dashed black line indicates the time at which the seizure was declared by the SOD

The proposed detector has a high average latency as depicted in Table 3; this might be caused by many reasons, such as delay in significant changes from the background EEG during a seizure. The brain’s electrical activity may take some time before showing a clear distinction between the background EEG and the ictal EEG. Also, the limited ability of CN and RPDE in capturing the early transition from normal to ictal state might cause high detection latency. Figure 6 illustrates this by showing the brain’s electrical activity of one channel (F7T7) during a seizure and the corresponding CN and RPDE plots. When the seizure starts at 300 s, there is a clear difference between the background and ictal activity. Yet, the detector declares the seizure after 27 s when there is a clearer distinction between the CN and RPDE of normal and ictal EEG.

Finally, to benchmark the detector, its performance is compared with other detectors in literature that used the same dataset. Table 4 lists the features used by other detectors, their performance, and the number of patients used to evaluate them. Specificity is computed for comparison, and it represents the percentage of non-seizure epochs that were correctly labeled as normal. Most of these detectors use a high number of features or complex post-processing procedures. When compared to these SODs, the proposed SOD has a higher sensitivity and a comparable FP rate. However, the detector needs to improve in terms of detection latency. Overall, the proposed SOD is attractive in terms of simplicity, sensitivity, and FP rate. It is worth noting that the sensitivity of the proposed detector was 100% as a trade-off for larger FP rates and higher latency.

**Table 4:** Comparison with other detectors in literature

Reference	Features	Sensitivity	FP rate	Specificity	Latency	No. of patients
[4]	Amplitude	96.9%		97.5%	3.60	7
[5]	Amplitude + CSP	100%	1.17		7.02	7
[18]	Wavelet energy features + CSP	100%	1.2		7.28	7
[6]	Statistical features	83.5%		90.3%	3.43	24
[10]	Entropy	99%		100%		23
[11]	Temporal synchrony	98.5%	21.4%ª		8.59	23
[12]	CN + energy features	100%	2.1		2.80	5
[13]	Spectral energy features	96%	0.34		4.60	24
[14]		95.1%	10.31			24
[16]	Spectral + spatial features	98%		91%	< 10	23
[17]	Statistical wavelet-based features	98.40%	0.80		6.30	18
[19]	Energy features	96%	0.10		1.89	22
[20]	Entropy of wavelet coefficients	100%	2.16		3.14	10
[21]		96.40%		98.6%	1.70	14
[22]		98.50%			1.76	24
[23]	Wavelet coefficients + CSP + PCA	97.20%	0.64		1.10	20
[24]	Raw EEG + CNN	99.50%	0.12		8.07	23
[25]		90.60%			4.68	23
**Proposed SOD**	**CN + RPDE**	**100%**	**0.53**	**99.9%**	**9.73**	**10**

^a^Reported as a % from the total alarms

## Conclusion

In this work, seizure’s onset was detected using a patient-specific SOD. The SOD was trained using synchrony and chaos features of the EEG. Synchronization features were estimated using the CN while the RPDE measured periodicity to quantify chaoticity. Seizure’s onset was successfully detected using the features either individually or when they are fused. The proposed detector achieved a sensitivity of 100%, an FP rate of 0.53 per hour, and a latency of 9.73 s. This SOD can be utilized in offline seizure detection to minimize the burden of scanning long EEG data.

To improve the performance while maintaining interpretability, it is possible to consider different approaches. One possible way is to investigate a simple noise rejection technique to filter artifacts from the EEG signal. Additionally, to enhance detection of focal seizures, it is possible to employ channel selection for quantifying synchrony. Neuronal features that characterize the pre-ictal state can be studied and utilized to reduce the detection latency. To improve the detector’s overall performance, it is possible to investigate other uncorrelated features that reflect the brain’s electrical activity during the ictal state.

The proposed detector used neuronal behavior during a seizure, and it revealed that an SOD that uses one feature can be sufficient to detect seizure with comparable performance to other SODs. Hence, to maintain interpretability and performance, it is important to use medically relevant features rather than using as many features as possible. This is not only related to automatic seizure detection studies, but also to other biomedical signal processing research.

## Appendix. RPDE calculation

The RPDE was calculated using the following steps:

1. Converting EEG channel to phase space representation
  A single EEG channel $s$ with a sampling period $\Delta t$ and *N* samples is represented as
  1 $$s{=\left[{s}_{0} {s}_{\Delta t} {s}_{2\Delta t} \cdots {s}_{(\mathrm{N}-1)\Delta t}\right]}^{T}$$
  where $s$ is an $N\times 1$ vector and each element ${s}_{i\Delta t}$ is a scalar value representing the signal’s amplitude at $t=i\Delta t$. The signal is converted to its phase space representation $S$ by using time-delayed versions of the signal $s$. This is referred to as the time-delay embedding approach and it results in the following matrix:
  2 $$S=\left[\begin{array}{cc}\begin{array}{cc}s& {s}_{\tau }\end{array}& \begin{array}{cc}\cdots & {s}_{\left(d-1\right)\tau }\end{array}\end{array}\right]$$
  where $s$ is the original signal as in (1) and it starts at $t=0$ (the first element is ${s}_{0}$); signal ${s}_{\tau }$ starts at $t=\tau$ and so on.
  The parameter $\tau$ represents the time delay, in seconds, between the elements within the sequence, and $d$ is the embedding dimension and represents the dimension of the phase space. In this work, $\tau =0.02 s$ and $d=3$, and these values were determined experimentally. In the reconstructed phase space $S$, each row ${S}_{i}$ is a d-dimensional point in the phase space. The first row ${S}_{0}$ is the first point.
2. Quantifying the recurrence periods within the phase space
  In this step, the constructed phase space representation in (2) is used to estimate the RPDE for signal $s$. This is done by the following:
  a A ball with a radius $\varepsilon$ is defined around the first point ${S}_{0}$ in the phase space $S$. The value of $\varepsilon$ is chosen to be 0.01 as this gave a better performance compared to other values.
  b Starting from ${S}_{0}$, a trajectory is followed forward in time by visiting the subsequent points (${S}_{1}, {S}_{2},\dots$). The Euclidean distance between ${S}_{0}$ and the visited points is calculated. A point ${S}_{j}$ leaves the ball when $\Vert {S}_{0}-{S}_{j}\Vert >\varepsilon$, where $\Vert \cdot \Vert$ represents the Euclidean norm. After the point ${S}_{j}$ leaves the ball, the trajectory is continued to be traced until another point ${S}_{k}$ enters the ball again, i.e., $\Vert {S}_{0}-{S}_{k}\Vert <\varepsilon$. The recurrence time $T$ for point ${S}_{0}$ is the difference between the index of ${S}_{k}$ and ${S}_{0}$: $T=k-0=k.$ In general, the recurrence time for a point ${S}_{i}$ that returns at point ${S}_{j}$ is $T=j-i$.
  c The recurrence time for point ${S}_{0}$ is recorded.
  d Steps a to c are repeated for all the other rows in $S$. For each point ${S}_{i}$, the subsequent points (${S}_{i+1}, {S}_{i+2},\dots$) are visited to estimate the recurrence time.
  e The recorded recurrence times are used to produce a histogram of recurrence times $R(T)$; the histogram includes the frequency of each recurrence time $T$. The histogram is normalized to obtain the recurrence time probability density $P(T)$:
    3 $$P\left(T\right)=\frac{R(T)}{\sum_{i=1}^{{T}_{\mathrm{max}}}R(i)}$$
    where ${T}_{\mathrm{max}}$ is the maximum recorded recurrence time.
  f The recurrence time probability density, $P(T)$, is used to obtain the RPDE by the following equation:
    4 $$\mathrm{RPDE}=\frac{-\sum_{i=1}^{{T}_{\mathrm{max}}}P\left(i\right)\mathrm{ln}\,P(i)}{\mathrm{ln}\,{T}_{\mathrm{max}}}$$
